# Retraction Note: Gastroprotective effect of desmosdumotin C isolated from *Mitrella kentii* against ethanol-induced gastric mucosal hemorrhage in rats: possible involvement of glutathione, heat-shock protein-70, sulfhydryl compounds, nitric oxide, and anti-*Helicobacter pylori* activity

**DOI:** 10.1186/s12906-024-04475-5

**Published:** 2024-04-19

**Authors:** Heyam Mohamed Ali Sidahmed, Ainnul Hamidah Syahadah Azizan, Syam Mohan, Mahmood Ameen Abdulla, Siddig Ibrahim Abdelwahab, Manal Mohamed Elhassan Taha, A. Hamid A. Hadi, Kamal Aziz Ketuly, Najihah Mohd Hashim, Mun Fai Loke, Jamuna Vadivelu

**Affiliations:** 1 Department of Pharmacy, Faculty of Medicine, University of Malaya, Kuala Lumpur, 50603 Malaysia; 2 Department of Molecular Medicine, Faculty of Medicine, University of Malaya, Kuala Lumpur, 50603 Malaysia; 3 Department of Chemistry, Faculty of Science, University of Malaya, Kuala Lumpur, 50603 Malaysia; 4 Medical Research Centre, Jazan University, P.O. Box 114, Jazan, Saudi Arabia; 5 Department of Medical Microbiology, Faculty of Medicine, University of Malaya, Kuala Lumpur, 50603 Malaysia

**Retraction Note: BMC Complement Med Ther 13, 183 (2013)**

10.1186/1472-6882-13-183

The Editors retracted this article because of concerns regarding a number of figures presented in this work. These concerns call into question the article’s overall scientific soundness and its authorship. An investigation conducted after its publication discovered similarities between images in this work and images in [1] and [2],

- Panel D in Fig. 5 and panel B in Fig. 5 in [1];
- Panel D in Fig. 6 and panel E in Fig. 5 in [2];
- Panel F in Fig. 5 and panel E in Fig. 4 in [1];
- The bottom part of panel F in Fig. 5 and the top part of panel E in Fig. 4 in [2];

The Editors therefore no longer have confidence in the integrity of the research presented in this article. Jamuna Vadivelu and Mun Fai Loke agree with the retraction. The remaining authors have not responded to correspondence from the Publisher about the retraction.
